# Applications of Scanning Electron Microscopy Using Secondary and Backscattered Electron Signals in Neural Structure

**DOI:** 10.3389/fnana.2021.759804

**Published:** 2021-12-02

**Authors:** Daisuke Koga, Satoshi Kusumi, Masahiro Shibata, Tsuyoshi Watanabe

**Affiliations:** ^1^Department of Microscopic Anatomy and Cell Biology, Asahikawa Medical University, Asahikawa, Japan; ^2^Department of Morphological Sciences, Kagoshima University Graduate School of Medical and Dental Sciences, Kagoshima, Japan

**Keywords:** osmium maceration method, section-face imaging, semithin section SEM, CLEM, CLSEM, serial section SEM, 3D, SEM

## Abstract

Scanning electron microscopy (SEM) has contributed to elucidating the ultrastructure of bio-specimens in three dimensions. SEM imagery detects several kinds of signals, of which secondary electrons (SEs) and backscattered electrons (BSEs) are the main electrons used in biological and biomedical research. SE and BSE signals provide a three-dimensional (3D) surface topography and information on the composition of specimens, respectively. Among the various sample preparation techniques for SE-mode SEM, the osmium maceration method is the only approach for examining the subcellular structure that does not require any reconstruction processes. The 3D ultrastructure of organelles, such as the Golgi apparatus, mitochondria, and endoplasmic reticulum has been uncovered using high-resolution SEM of osmium-macerated tissues. Recent instrumental advances in scanning electron microscopes have broadened the applications of SEM for examining bio-specimens and enabled imaging of resin-embedded tissue blocks and sections using BSE-mode SEM under low-accelerating voltages; such techniques are fundamental to the 3D-SEM methods that are now known as focused ion-beam SEM, serial block-face SEM, and array tomography (i.e., serial section SEM). This technical breakthrough has allowed us to establish an innovative BSE imaging technique called section-face imaging to acquire ultrathin information from resin-embedded tissue sections. In contrast, serial section SEM is a modern 3D imaging technique for creating 3D surface rendering models of cells and organelles from tomographic BSE images of consecutive ultrathin sections embedded in resin. In this article, we introduce our related SEM techniques that use SE and BSE signals, such as the osmium maceration method, semithin section SEM (section-face imaging of resin-embedded semithin sections), section-face imaging for correlative light and SEM, and serial section SEM, to summarize their applications to neural structure and discuss the future possibilities and directions for these methods.

## Introduction

Scanning electron microscopy (SEM) enables images to be obtained by detecting various signals [e.g., secondary electrons (SEs), backscattered electrons (BSEs), X-ray, and cathodoluminescence)] that escape from specimens when an incident electron probe emitted from an electron gun strikes the observation targets. Among these signals, both SE and BSE signals are most commonly used in biological and biomedical research. SEs are emitted near the surface of specimens and provide surface information on tissues and cells. SEs that are produced in deeper regions of the samples are absorbed because of their low energy. More SEs escape from projections, such as microvilli than from the flat surface of specimens. This phenomenon is called the edge effect, which contributes to the image formation of the three-dimensional (3D) surface topography of specimens; the fine protrusion of the cytoplasm appears bright on the image, whereas flat areas appear dark. Various SEM techniques that use SE signals for bulk specimens have been developed by numerous SEM researchers, which include the conventional technique as well as hydrochloric acid digestion (Evan et al., 1976), sodium hydroxide maceration (Takahashi-Iwanaga and Fujita, 1986), and potassium hydroxide maceration (Ushiki and Ide, 1987), methods for the removal of connective tissue, the alkali-water maceration method for revealing the collagen fibrillar network (Ohtani, 1987), vascular corrosion casting for visualizing the microvascular circulation (Murakami, 1971), and the osmium maceration method for observing the subcellular structure (Tanaka and Naguro, 1981; Tanaka and Mitsushima, 1984). A common feature of these SEM methods is the removal or digestion of unnecessary structures from bulk specimens for visualizing the structures of interest in tissues.

In contrast to SEs, BSEs can escape from deeper regions of specimens and contribute to the depth images of tissues and cells owing to their high energy. The production of BSEs is heavily dependent on the mean atomic number of the specimen, and it allows the detection of composition information. Because more BSEs are emitted from heavy metals than bio-specimens with a small average atomic number, the osmium, uranium, and lead, which are commonly used as fixatives or staining, appear brighter in images compared with tissues. BSE signals have been used to visualize gold particles in bulk specimens labeled using immunogold techniques (Kariya et al., 2016) and heavy metals impregnated in tissues prepared using histochemical techniques (Koga et al., 2016a). Thus, SEM preparation techniques for bulk samples in biological and biomedical research are diverse, albeit challenging.

With recent advances in SEM instruments (electron guns, lenses, and signal detection systems), imaging of both resin-embedded tissue blocks (block-face imaging) and resin-embedded sections (section-face imaging) is possible with SEM using a BSE detector (BSE-mode SEM). These novel BSE imaging techniques have broadened the biological applications of SEM and have been applied to recent 3D-SEM techniques, such as serial block-face SEM (SBF-SEM; Denk and Horstmann, 2004), focused ion beam SEM (FIB-SEM; Ichimura et al., 2015), and serial section SEM, which are attracting considerable attention in the microscopy, biological, and biomedical fields. SBF-SEM and FIB-SEM are based on block-face imaging, whereas serial section SEM is based on section-face imaging.

This article aims to highlight the possibilities of useful SEM techniques for examining bio-specimens, which include the osmium maceration method using SE-mode SEM and section-face imaging and serial section SEM using BSE-mode SEM. Unlike novel 3D-SEM techniques that use BSE-mode SEM, the osmium maceration method is a pivotal imaging technique for SE-mode SEM that provides direct 3D visualization of the intracellular structure without the need for 3D reconstruction (Tanaka and Naguro, 1981; Tanaka and Mitsushima, 1984; Koga and Ushiki, 2006). Using the maceration method, the 3D surface texture of organelles, such as the Golgi apparatus, smooth and rough endoplasmic reticulum (ER), mitochondria, nuclei, and ribosomes, can be effectively detected on the freeze-cracked surface of cells that have been treated for several days with diluted osmium tetroxide (OsO_4_). Section-face imaging of resin-embedded semithin sections (i.e., semithin section SEM) using BSE-mode SEM provides high-resolution ultrathin images that are comparable to conventional transmission electron microscopy (TEM) images of ultrathin sections without requiring ultramicrotomy (Koga et al., 2015b). The combination of section-face imaging with the Tokuyasu cryo-sectioning method, which was recently developed by our group, is an original correlative light and scanning electron microscopy (CLSEM) method that enables the visualization and localization of target molecules tagged with fluorescence dyes at an electron microscopy (EM) resolution (Kusumi et al., 2018). Serial section SEM is a novel 3D-SEM technique to image consecutive ultrathin sections embedded in resin on solid substrates and is used to generate 3D reconstruction models of cells and organelles from sequential tomographic images captured by BSE-mode SEM (Horstmann et al., 2012; Reichelt et al., 2012; Koga et al., 2016b). In this article, we introduce the various SEM methods that use SE (for the osmium maceration method) and BSE (for section-face imaging and serial section SEM) signals and discuss their applications in the morphological analysis of neural tissues and the future directions for SEM analysis on bio-specimens.

## Materials and Methods

### Animals

Ten male adult Wistar rats purchased at 8 weeks of age from Sankyo Laboratory Service Co., Ltd. (Tokyo, Japan) were used in accordance with the Guidelines for the Care and Use of Laboratory Animals (Institute of Laboratory Animal Resources; National Research Council, Washington, DC, 1996) under the permission of the experimental animal welfare committee of Asahikawa Medical University.

### Preparation for Osmium Maceration Method

**Figure 1:**
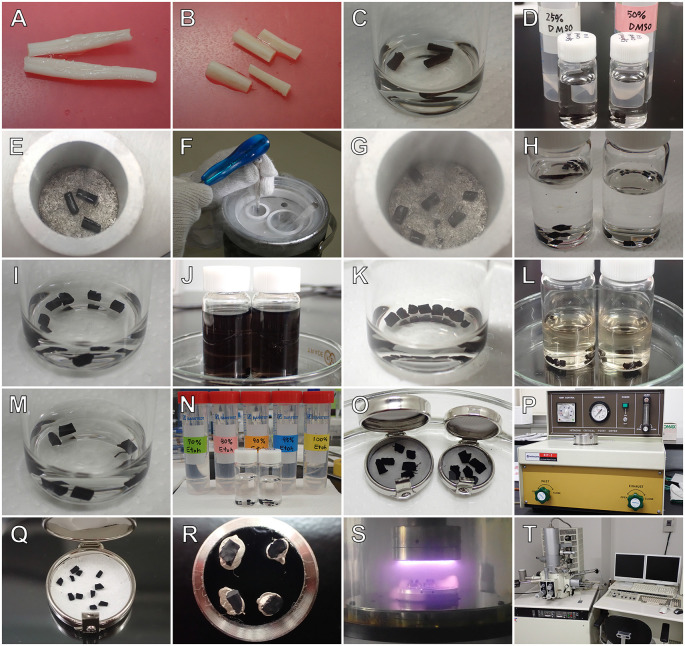
Outline of the osmium maceration method. After perfusion fixation, tissues are removed **(A)**, cut into small pieces **(B)**, and further fixed with osmium tetroxide (OsO_4_) **(C)**. They are then immersed in 50% dimethyl sulfoxide (DMSO) solution **(D)**, frozen on an aluminum plate precooled with liquid nitrogen **(E)**, and freeze-cracked into two pieces using a screwdriver and hammer **(F)**. The freeze-cracked pieces **(G)** are placed back into the DMSO solution for thawing **(H)** and refixed with OsO_4_ **(I)**. For cell maceration, specimens are immersed in 0.1% OsO_4_ for several days **(J)**. The macerated tissues are further fixed with OsO_4_ **(K)**, treated with 1% tannic acid **(L)**, placed in 1% OsO_4_ **(M)**, dehydrated in a series of graded ethanol **(N)**, transferred to isoamyl acetate **(O)**, and dried in a critical point dryer (HCP-2; Hitachi) **(P)**. Dried specimens **(Q)** are mounted onto SEM holders with silver paste **(R)**, coated with platinum-palladium in an ion-sputter coater (E1030; Hitachi) **(S)**, and observed in a field emission scanning electron microscope (S-4100; Hitachi) **(T)**.

The workflow for the osmium maceration method, which is a modification of the original protocol by our group, is summarized in Figure 1. Briefly, animals under deep anesthesia were perfused with a mixture of 0.5% glutaraldehyde (GA) and 0.5% paraformaldehyde (PFA) in 0.1 M phosphate buffer (PB; pH 7.4). A relatively diluted aldehyde fixative (i.e., a mixture of 0.5% GA and 0.5% PFA) is most suitable for removing cytoplasmic matrices from the fractured surface of cells using the osmium maceration procedure (Tanaka and Mitsushima, 1984). After perfusion fixation, tissues were excised from animals, cut into small pieces, and postfixed with 1% OsO_4_ (0.1 M PB). Specimens were then cryoprotected in 50% dimethyl sulfoxide (DMSO), frozen on an aluminum plate that was precooled with liquid nitrogen, and cracked into two pieces using a screwdriver and hammer. Several pieces of tissues were subsequently thawed in the DMSO solution. To remove soluble cytoplasmic proteins from the cracked surface of cells, the tissues were immersed in 0.1% OsO_4_ (0.1 M PB) at 20°C for several days. The macerated tissues were then processed for conductive staining; the specimens were immersed in 1% OsO_4_ solution, treated with 1% tannic acid, and further soaked in 1% OsO_4_. After conductive staining, they were dehydrated in a series of graded ethanol, transferred to isoamyl acetate, and dried in a critical point dryer (HCP-2; Hitachi, Tokyo, Japan). The dried specimens were mounted onto aluminum bases, coated with platinum-palladium in an ion-sputter coater (E1030; Hitachi), and observed through a scanning electron microscope (S-4100; Hitachi). Scanning electron microscopes that are equipped with field emission (FE) guns provide sufficient information on the 3D ultrastructure of macerated tissues. Details of the protocol for the osmium maceration method are available in previous publications (Tanaka and Naguro, 1981; Tanaka and Mitsushima, 1984; Koga and Ushiki, 2006).

### Preparation for Section-Face Imaging of Semithin Sections Embedded in Resin Using BSE-Mode (Semithin Section) SEM

**Figure 2:**
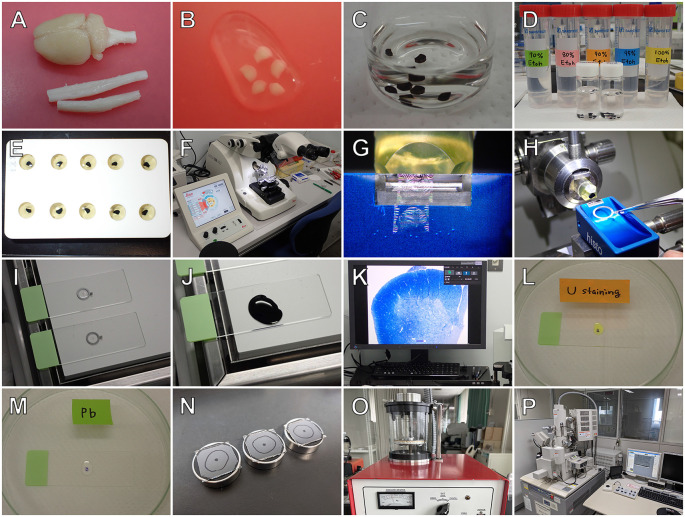
Outline of semithin section SEM. After perfusion fixation, tissues are removed **(A)**, cut into small pieces **(B)**, postfixed with osmium tetroxide **(C)**, dehydrated with a graded series of ethanol **(D)**, and embedded in epoxy resin **(E)**. Resin-embedded semithin sections (500-nm thickness) are cut using a Histo knife (Diatome) **(G)** in an ultramicrotome (EM UC-7; Leica) **(F)**, picked up with an aluminum loop (Transfer Ring; Microstar) **(H)**, attached on glass microscope slides by heating on a hot plate **(I)**, and stained with toluidine blue **(J)**. The semithin sections are observed using light microscopy (LM) **(K)**, and the regions of interest are photographed using a charge coupled device camera (DP27; Olympus). The sections are subsequently stained with heavy metals of uranyl acetate **(L)** and lead citrate **(M)**, mounted onto aluminum stubs **(N)**, and coated with carbon using a carbon coater (VC-100S; Vacuum Device) **(O)**. Finally, the same area of the sections that was previously photographed using LM is observed using a scanning microscope with a backscattered electron detector (SU70; Hitachi) **(P)**.

The workflow for semithin section SEM established by our group is summarized in Figure 2. Under deep anesthesia, animals were perfused with an aldehyde fixative, and tissues were excised, cut into small pieces, post-fixed with 1% OsO_4_, dehydrated with a graded series of ethanol, embedded in epoxy resin, and polymerized in an incubator at 60°C for 48 h. Resin blocks were then trimmed to an appropriate size, and 500-nm thickness semithin sections were cut with an ultramicrotome (EM UC-7; Leica, Wetzlar, Germany) using a diamond knife (Diatome, Biel, Switzerland). Subsequently, these sections were picked up using aluminum loops (Transfer ring; Micro Star Co., Tokyo, Japan) and mounted on glass microscope slides. Semithin sections were adhered to the glass slides by heating on a hot plate (60°C) and stained with toluidine blue, and the regions of interest (ROI) of tissues were photographed using light microscopy (LM). Following LM observation, sections were stained with heavy metals (i.e., uranyl acetate and lead citrate), and the sections on the glass slides were mounted onto aluminum bases. The sections were then coated with carbon using a carbon coater (VC-100S, Vacuum Device, Ibaraki, Japan), and the same regions of the specimens previously photographed using LM were acquired using BSE-mode SEM under an accelerating voltage of 2–2.5 kV (SU-70; Hitachi). The black and white reversed images of the resin-embedded semithin sections captured by BSE-mode SEM resemble TEM images of ultrathin sections. Detailed protocols for semithin section SEM are provided in our original article (Koga et al., 2015b).

### Combination of the Cryo-Sectioning Technique With Section-Face Imaging

**Figure 3:**
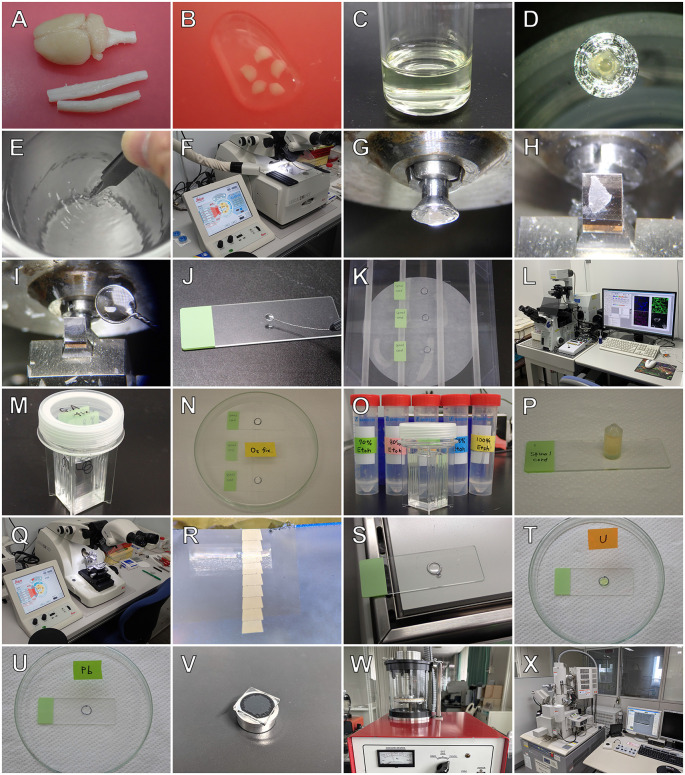
Outline of the combination of the Tokuyasu cryo-sectioning method with section-face imaging. After perfusion fixation, tissues are removed from animals **(A)**, cut into small pieces **(B)**, and immersed in cryoprotectant materials **(C)**. The specimens are then mounted on sample holders **(D)**, rapidly frozen in liquid nitrogen **(E)**, and placed into a cryo-chamber attached to an ultramicrotome (EM FC-7; Leica, **F,G**). Semithin cryosections of tissues are cut using a diamond knife (Diatome) **(H)** in a low-temperature sectioning system. The cryosections are picked up with a 2.3 M sucrose droplet on a wire loop **(I)** and mounted on glass microscope slides for subsequent immunocytochemistry **(J)**. After immunocytochemical staining **(K)**, regions of interest of the cryosections are photographed with a confocal laser scanning microscope (FV1000-D; Olympus) **(L)**. The cryosections are then further fixed with glutaraldehyde **(M)** and osmium tetroxide **(N)**, dehydrated with a graded series of ethanol **(O)**, and embedded in epoxy resin **(P)**. Ultrathin sections are cut from the resin-embedded semithin cryosections with an ultramicrotome **(Q)** using a diamond knife (Diatome) **(R)**, mounted onto glass slides **(S)**, stained using heavy metals of uranyl acetate **(T)** and lead citrate **(U)**, mounted onto aluminum stubs **(V)** and coated with carbon in a carbon coater (VC-100S; Vacuum Device) **(W)**. The same area imaged previously using laser microscopy is observed using backscattered electron-mode scanning electron microscopy (SU70; Hitachi) **(X)**.

The workflow for the combination of the Tokuyasu cryo-sectioning technique with section-face imaging method is summarized in Figure 3. Briefly, small pieces of tissue prefixed with aldehyde fixatives by perfusion were immersed in cryoprotectant materials [i.e., a mixture of 20% polyvinylpyrrolidone (PVP) and 1.84 M sucrose solution], placed on mounting blocks, rapidly frozen in liquid nitrogen, and placed into a cryo-chamber attached to an ultramicrotome (EM UC-7; Leica). Semithin cryosections with a thickness of 0.5–1 μm were cut from the frozen tissues using a diamond knife (Diatome), picked up using 2.3 M sucrose droplets on wire loops, and attached to glass microscope slides. They were then rinsed with phosphate buffered saline (PBS) and incubated with 2% normal donkey serum in PBS for 30 min at 20°C, followed by incubation with a mixture of primary antibodies of different species [a sheep polyclonal anti-trans-Golgi network (TGN)38 antibody (AbD Serotec, Oxford, UK) and a goat polyclonal anti-choline acetyltransferase antibody (Bioss antibodies, MA, USA] for 12 h at 4°C. Subsequently, sections were thoroughly rinsed in PBS, incubated with a mixture of secondary antibodies [Alexa Fluor 488-labeled anti-sheep immunoglobulin G (IgG) and Alexa Fluor 594-labeled anti-goat IgG (Invitrogen, CA, USA] for 1 h at 4°C, washed with PBS, and coverslipped in SlowFade®ledR Diamond Antifade with 4’, 6-diamidino-2-phenylindole (Invitrogen). Immunostained sections were then observed with a confocal laser scanning microscope (FV 1000-D; Olympus, Tokyo, Japan). After LM observation of the ROIs, the cryosections were refixed with GA, postfixed with osmium tetroxide, stained with uranyl acetate, and embedded in epoxy resin. Ultrathin sections of cryosections embedded in resin are cut with an ultramicrotome (EM UC-7; Leica) using a diamond knife (Diatome), mounted on glass slides, and coated with carbon. Finally, the same regions observed using LM were examined using BSE-mode SEM (SU-70; Hitachi), and correlative microscopy was performed by superimposing the LM and SEM images. Detailed information on this CLSEM is available in our previous report (Kusumi et al., 2018).

### Preparation for Serial Section SEM and the 3D Reconstruction Method

**Figure 4:**
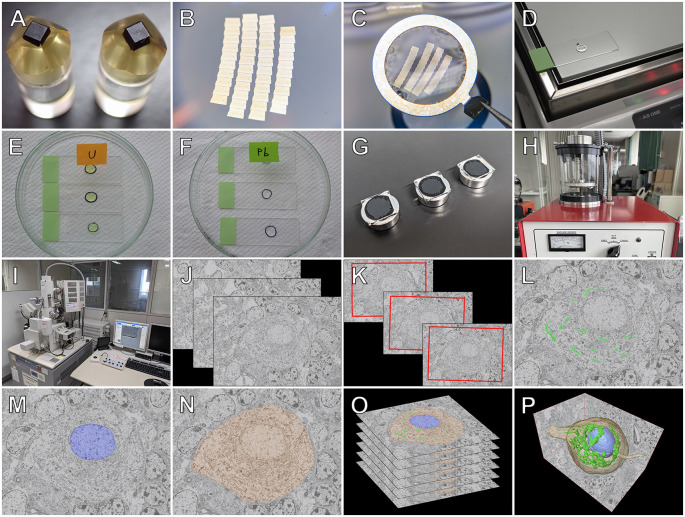
Workflow for serial section SEM. After perfusion fixation, tissues are further fixed with osmium tetroxide and embedded in resin **(A)**. Several arrays of ribbons with serial ultrathin sections (thickness of 80–100 nm) are cut using a diamond knife (Diatome, **B**), picked up with an aluminum loop (Transfer Ring; Microstar) **(C)**, and attached to glass microscope slides by heating on a hot plate **(D)**. Several hundred serial sections are obtained by repeating cycles of the sectioning procedure. The serial sections are stained with heavy metals of uranyl acetate **(E)** and lead citrate **(F)**, and the glass slides with the serial sections are mounted onto aluminum stubs **(G)** and coated with carbon using a carbon coater (VC-100S; Vacuum Device) **(H)**. Regions of interest of the serial sections are imaged using scanning electron microscopy with a backscattered electron (BSE) detector (SU70; Hitachi) **(I)**. For three-dimensional (3D) reconstruction, BSE images of the serial sections **(J)** are aligned **(K)**, and the contours of the structures of interest, such as the Golgi apparatus **(L)**, nucleus **(M)**, and cytoplasm **(N)**, are segmented and stacked **(O)**. Finally, 3D surface rendering images of the regions of interest are generated **(P)**.

The workflow for serial section SEM that we established is summarized in Figure 4. Briefly, serial ultrathin sections (section thickness: 80–100 nm) of resin-embedded tissues were cut into single ribbons with an ultramicrotome (EM UC-7; Leica) using a diamond knife (Diatome). Ribbons containing 10–15 consecutive ultrathin sections were created, and several aligned ribbons (50–100 sections in total) were picked up using an aluminum loop (Transfer ring; Micro Star) and transferred onto a glass microscope slide. Several hundred to a thousand serial ultrathin sections were mounted on glass slides *via* repeated cycles of this sectioning procedure, stained with heavy metals (i.e., uranyl acetate and lead citrate), and coated with carbon for conductive treatment. The ROI of the serial sections was then imaged using BSE-mode SEM (SU-70; Hitachi). For the 3D reconstruction of the structures of interest, serial tomographic images acquired using SEM were loaded into the Amira software (Thermo Fisher Scientific, MA, USA) and aligned using the align tool. The aligned images were subsequently imported into the Photoshop software (Adobe Systems Inc., CA, USA), and the structures of interest (e.g., the Golgi apparatus, ER, mitochondria and, nuclei) were segmented by delineating their boundary contours. The segmented images were then reimported into Amira, and 3D surface rendering images were created. Detailed information on serial section SEM and the 3D reconstruction method is available in our original articles (Koga et al., 2016b; Koga et al., 2017b).

## Results

### Application of the Maceration Method for Nerve Tissues

**Figure 5:**
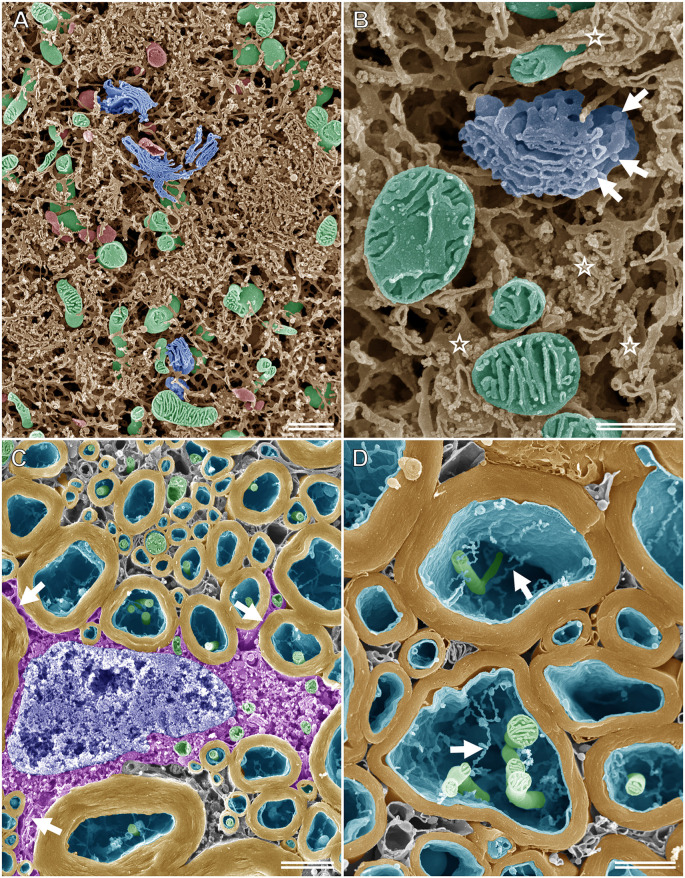
SEM micrographs of nerve cells and tissues after performing the osmium maceration method. **(A)** Intracellular structure of the rat spinal ganglion cell showing part of the soma. The three-dimensional structure of the Golgi apparatus (blue), mitochondria (green), lysosomes (red), and endoplasmic reticulum (ER; brown) in the soma of the rat spinal ganglion cell observed using SE-mode SEM. **(B)** High magnification image of the Golgi apparatus (blue), mitochondria (green), and ER (brown) in the soma of the rat ganglion cell. Ribosomes can be recognized on the cisterns of the flat ER sheets (stars). Arrows: vesicles attached on the periphery of the Golgi cisterns. **(C)** The myelin sheath (yellow), axons (cyan), and oligodendrocytes (magenta) in the white matter of the rat spinal cord on the freeze-cracked surface. Arrows: processes of the oligodendrocytes, which arise from the cell body. Green: mitochondria, blue: nucleus. **(D)** Closer view of the myelin sheath (yellow). Both mitochondria (green) and tubular-shaped membranes (arrows) in the axon (cyan) are clearly visible. **(A)** Scale bar: 1 μm; **(B)** scale bar: 500 nm; **(C)** scale bar: 1.5 μm; **(D)** scale bar: 1 μm.

When specimens prepared for the osmium maceration method are examined using high-resolution FE-SEM, the 3D ultrastructure of organelles can be observed on the fractured surface of tissues, much like the schematic drawings found in textbooks (Tanaka and Naguro, 1981; Tanaka and Mitsushima, 1984; Koga and Ushiki, 2006). Figure 5A shows an SEM image of the rat spinal ganglion cell prepared according to the osmium maceration method. The 3D structure of organelles, such as the ER in a Nissl body, Golgi apparatus, mitochondria, and lysosomes could be seen clearly in the soma. Osmium maceration allows the observation of structures that are present in deeper regions on the fractured plane of specimens because cytoplasmic soluble proteins are extracted with diluted OsO_4_ after freeze-cracking treatment during the osmium maceration procedure. High-resolution SEM provided detailed information on the 3D morphological organization of these organelles and detected the spatial relationship among the Golgi, ER, and mitochondria in the ganglion cells (Figure 5B). The tubular and sheet morphology of the ER and mitochondria with plate-like cristae in the matrix space could be seen around the Golgi apparatus, with several flat cisterns piled up in layers. Both tubular and sheet-shaped ER were connected to form the spatially complicated and continuous network. Ribosomes were found on the cisterns of the flat ER sheets, and vesicles attached to the periphery of the Golgi cisterns were also clearly recognizable under high-resolution SEM. An SEM micrograph of the myelin sheath, axons, and oligodendrocytes in the white matter of the rat spinal cord is displayed in Figure 5C. Processes arising from the cell body of the oligodendrocyte, which was located around the myelin sheath, could be identified on the cracked surface of the osmium-macerated tissue. High-magnification images enabled further visualization of the ultrastructure of the myelin sheath and the inside of axons; the 3D spatial relationship between the mitochondria and the fine thread-like membrane structures (i. e., narrow anastomosed tubules of smooth ER) could be seen clearly within the axon (Figure 5D). Thus, the osmium maceration method allows the direct observation of the 3D morphological feature of organelles in nerve tissues, which include the soma of nerve cells, myelin, axons, glial cells, and Schwann cells, especially their surface texture and spatial organization (Koga and Ushiki, 2006; Takahashi-Iwanaga and Iwanaga, 2011; Nomura et al., 2013).

### Application of Semithin Section SEM for Nerve Tissues

**Figure 6:**
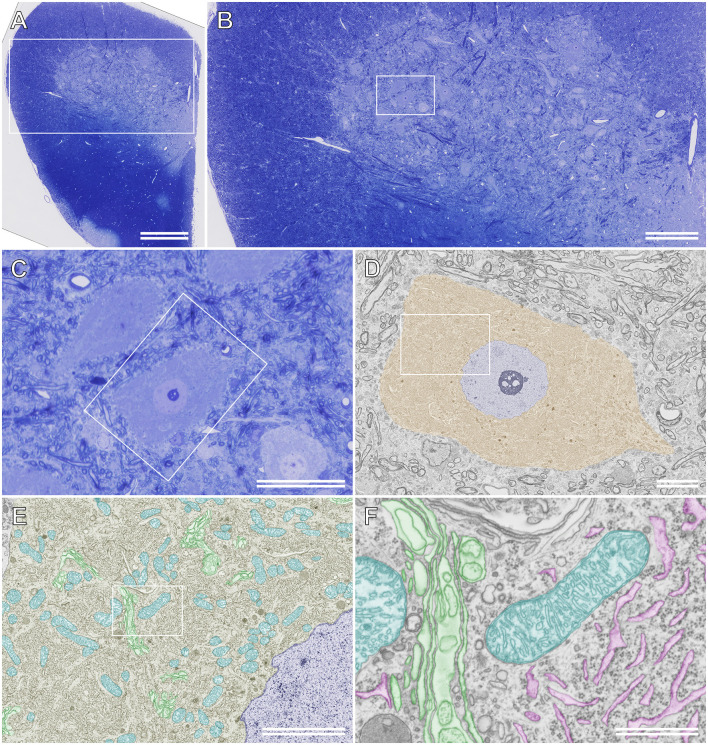
Semithinsection of the rat spinal cord observed using light microscopy (LM)and scanning electron microscopy (SEM). **(A)** A whole image ofthe resin-embedded semithin section observed using LM. The section isstained with toluidine blue. The boxed area is enlarged and shown in**(B)**. **(B)** LM images of the semithin section of the boxed area in **(A)**. The boxed area is enlarged and shown in **(C)**. **(C)** LM image of the semithin section of the boxed area in **(B)**. **(D)** The boxed area in **(C)** is observed using backscattered electron (BSE)-mode SEM. Brown: soma of the motor neuron, blue: nucleus. The boxed area is enlarged and shown in **(E)**. **(E)** Closer view of the motor neuron. Blue: nucleus, cyan: mitochondria, green: Golgi apparatus. The boxed area is enlarged and shown in **(F)**. **(F)** High-resolution image of the motor neuron. The ultrastructure of the Golgi apparatus (green), rough ER (magenta), and mitochondria (cyan) were imaged using BSE-mode SEM. **(A)** Scale bar: 500 μm; **(B)** scale bar: 200 μm; **(C)** scale bar: 50 μm; **(D)** scale bar: 10 μm; **(E)** scale bar: 5 μm; **(F)** scale bar: 1 μm.

Because semithin section SEM provides ultrathin information on resin-embedded semithin sections using BSE-mode SEM, high-resolution images of structures of interest can be obtained from the same sections after comprehensive observation using LM in advance. Furthermore, unlike TEM, BSE-mode SEM enables the observation of sections on glass microscope slides without the obstruction of the mesh grid, which may hide vital biological phenomena in specimens. Figure 6D shows an ultrathin BSE image of the soma of the rat motor neuron in a semithin section that was photographed beforehand using LM. The ultrastructure of organelles, such as the Golgi apparatus, ER, and mitochondria, was recognized clearly using BSE-mode SEM, even though the semithin sections on the glass slides were stained with toluidine blue (Figures 6E,F).

Detailed imaging of subcellular structures requires scanning electron microscopes equipped with FE-guns and high-sensitivity BSE detectors rather than conventional scanning electron microscopes equipped with tungsten filament guns, which include those that include a high-sensitivity detector. Additionally, the positional relationship between the specimen and the objective lens is a crucial factor for high-resolution observation. There are three types of objective lenses: the conventional “out-lens-type,” the “semi-in-lens (snorkel lens) type,” and the “in-lens-type.” Scanning electron microscopes equipped with a snorkel lens are most suitable for the seamless analysis of resin-embedded sections from low to high magnification.

The conductive treatment of metal coating is critical to the preclusion of charging artifacts during SEM observations of resin-embedded sections mounted on glass slides. Platinum and platinum-palladium, which are targets for metal coating, are usually used for SE-mode SEM of balk specimens, but they are unsuitable for high magnification observations because their particles are visible using BSE-mode SEM under >50,000-fold (Koga et al., 2015b). Thus, we commonly coat the resin-embedded sections with carbon or osmium particles, which are less prominent under BSE observation at higher magnifications. We have rarely encountered the distortion or split of sections coated with carbon or osmium using electron-beam damage. Resin-embedded sections mounted on conductive substrates, such as silicon wafers, instead of glass slides do not require coating materials, such as carbon, osmium, or platinum (Horstmann et al., 2012).

Semithin section SEM requires no highly skilled techniques, such as ultramicrotomy, and enables the acquisition of sufficient information on the fine structure of resin-embedded semithin sections that is equivalent to that obtained using TEM of ultrathin sections. As shown in Figure 6, this innovative technique is also useful for correlating LM and EM information obtained from the same sections.

### Combination of the Cryo-Sectioning Technique With Section-Face Imaging

**Figure 7:**
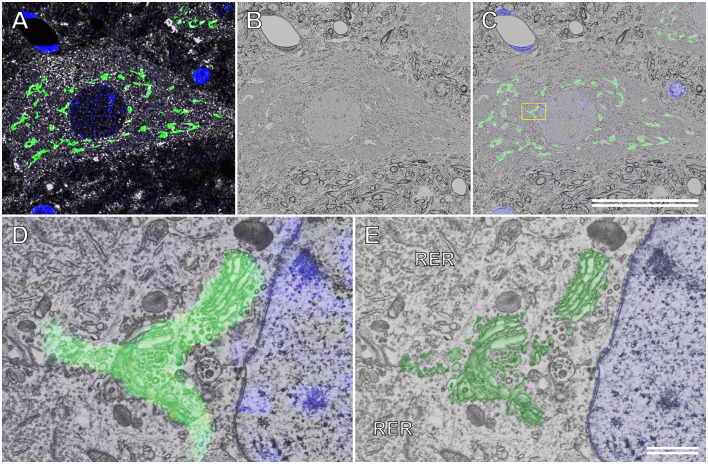
Correlative immunofluorescence and backscattered electron (BSE) images of a motor neuron in the rat spinal cord. **(A)** Immunofluorescence image of a semithin cryosection immunocytochemically stained with sheep polyclonal anti-TGN38 (green pseudocolor) and goat polyclonal anti-choline acetyltransferase (white pseudocolor) antibodies. Nuclei are also labeled using 4’,6-diamidino-2-phenylindole (blue pseudocolor). **(B)** BSE image of an ultrathin section cut from the semithin cryosection embedded in resin. The BSE micrograph is of the same area as that shown in **(A)**. **(C)** The immunofluorescence image in **(A)** is overlapped with the BSE image in **(B)**. The boxed area is enlarged and shown in **(D)**. **(D)** The higher magnification image demonstrates that the fluorescence dyes that indicate the Golgi-associated protein (TGN38) have appropriately localized the Golgi apparatus. **(E)** The BSE image eliminating the fluorescence image from the merged image in **(D)**. The profile of the ultrastructure of the Golgi apparatus (green) corresponds to the fluorescence image of TGN38 in **(D)**. Blue: nucleus. **(C)** Scale bar: 50 μm; **(E)** scale bar: 1 μm.

Figure 7A shows the immunocytochemical localization of the Golgi-associated protein, TGN38, and the motor neuronal marker, choline acetyltransferase (ChAT), in a semithin cryosection of the rat spinal cord. The Golgi apparatus was located throughout the soma of the motor neuron. This ultrathin section image of the resin-embedded cryosection of the motor neuron was observed using BSE-mode SEM (Figure 7B). The localization of TGN38 was mapped by superposing the immunofluorescence image onto the corresponding BSE image (Figure 7C). This merged image was enlarged to localize TGN38 labeled with fluorescence dyes at the EM resolution level, and the fluorescence signal for TGN38 could be detected on the fine structure of the Golgi apparatus (Figure 7D). The well-preserved ultrastructure of the Golgi apparatus, as well as the ER and its ribosomes, could be observed in the soma of the spinal ganglion cell prepared using the CLSEM method (Figure 7E).

### Application of Serial Section SEM for the Purkinje Cell

**Figure 8:**
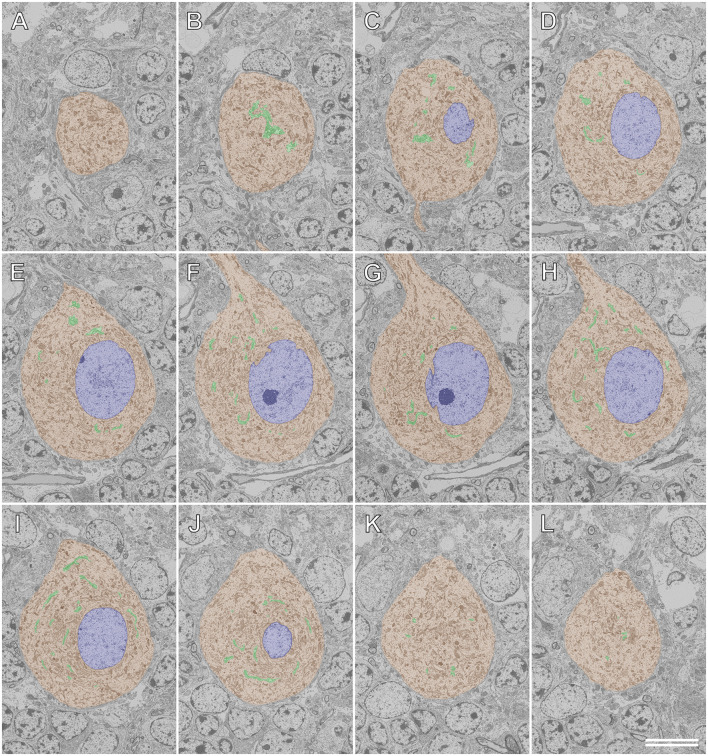
Serial backscattered electron images of ultrathin sections of a Purkinje cell in the rat cerebellum. Section numbers 20 **(A)**, 40 **(B)**, 60 **(C)**, 80 **(D)**, 100 **(E)**, 120 **(F)**, 140 **(G)**, 160 **(H)**, 180 **(I)**, 200 **(J)**, 220 **(K)**, and 240 **(L)** from a set of 281 serial ultrathin sections are shown. The Golgi apparatus, nucleus, and soma of the Purkinje cell are pseudocolored green, blue, and brown, respectively. Scale bar: 10 μm.

**Figure 9:**
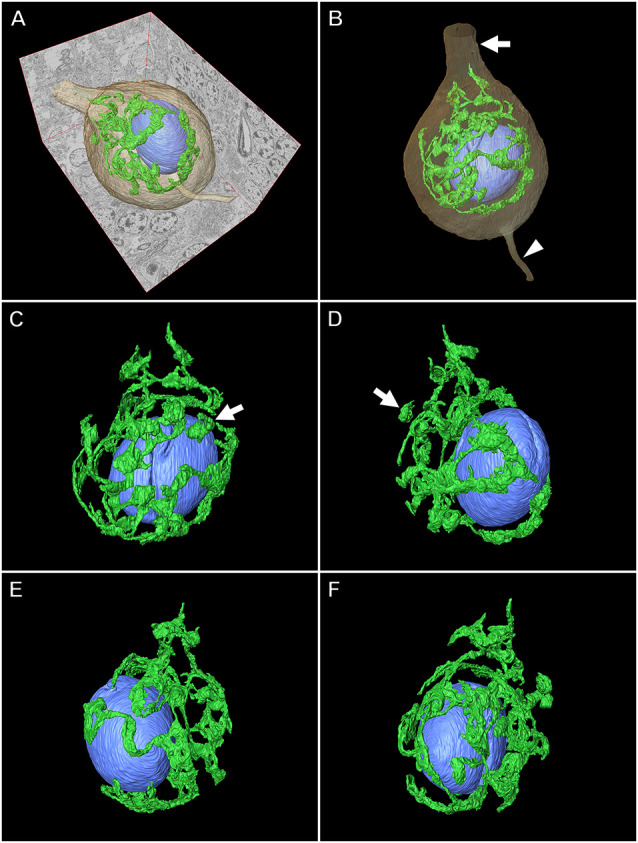
Three-dimensional (3D) reconstruction image of a Purkinje cell in the rat cerebellum. **(A,B)** Surface rendering models of the Golgi apparatus (green), nucleus (blue), and soma (brown) of the Purkinje cell created from 281 serial tomographic images obtained using BSE-mode SEM. The arrow and arrowhead indicate the dendrite and axon, respectively. Various angle views of the 3D model of the Golgi apparatus and nucleus are shown in **(C)**, **(D)**, **(E)**, and **(F)**. The Golgi apparatus in the Purkinje cell comprises a principally continuous basket-like structure that surrounds the nucleus. Arrows show discontinuous stacks of the Golgi apparatus.

Figures 8, 9 illustrate the application of serial section SEM in the Golgi apparatus of a Purkinje cell in the rat cerebellum. Figures 8A–L represent serial tomographic images of the Purkinje cell, which were captured using SEM of serial ultrathin sections of the rat cerebellum on glass microscope slides. We required 281 slices of serial sections to create surface rendering images of the structures of interest, such as the soma, Golgi apparatus, and nucleus of the Purkinje cell. The 3D reconstruction images of the soma, Golgi apparatus, and nucleus of the Purkinje cell are shown in Figure 9. The Golgi apparatus surrounding the nucleus occupied the widest area of the soma and the Golgi stacks, the fundamental units of this organelle, and were connected to each other to form a basket-like structure; however, some of the stacks were separate from the main body (Koga et al., 2017b). These 3D surface rendering images shown in Figure 9 demonstrated that a section thickness of 80–100 nm was sufficient to reconstruct a 3D configuration of the entire Golgi apparatus. Our 3D model corresponds well to the silver-impregnated reticular apparatus in the Purkinje cell body of an owl that was reported by Golgi (1898, 1989). Using serial section SEM, we clarified that the Golgi apparatus is a single continuous mass in various cell types (e.g., epithelial principal cells in the epididymal duct, gonadotropes in the anterior pituitary gland, and pancreatic acinar cells), except for in Purkinje cells (Koga et al., 2016b, 2017b).

## Discussion

### Osmium Maceration Method: History and Significance

Conventional preparation methods for bulk specimens using SE-mode SEM have been applied to analyze the 3D surface topography of tissues and cells. To visualize the intracellular structure using SE-mode, SEM researchers have introduced various cracking techniques, such as ethanol and DMSO freeze fracturing (Tokunaga et al., 1974) and styrene resin cracking (Tanaka et al., 1974) methods. However, these techniques are insufficient for exposing the subcellular structure buried in the cytosolic matrices; only a mirror-like surface can be recognized on freeze- or resin-cracked surfaces of tissues. Tanaka and colleagues (Tanaka and Naguro, 1981; Tanaka and Mitsushima, 1984) developed innovative preparation techniques for observing the 3D structure of cell organelles using SEM without the need for any reconstruction processes, which were designated as osmium maceration methods (i.e., the osmium-DMSO-osmium [O-D-O] method and the aldehyde-osmium-DMSO-osmium [A-O-D-O] method) and led to the novel use of SEM for examining bio-specimens. In the maceration methods, cytoplasmic matrices in the freeze-cleavage plane of cells, which are directly fixed with OsO_4_ by immersion (i.e., the O-D-O method) or fixed by perfusion with an aldehyde fixative and immersion with OsO_4_ (i.e., the A-O-D-O method), are removed by soaking tissues in diluted OsO_4_ for several days. This preparation step is termed the osmium maceration procedure, which is a pivotal process in selectively maintaining the membranous organelles in the cells. Usually, a high-concentration solution (1%–2%) of OsO_4_ is used as the fixative for cell membranes. It is well established that OsO_4_ reacts with lipid molecules of membrane components. However, it is less known that diluted OsO_4_ also has the potential for removing cytoplasmic soluble proteins from the cytoplasm.

In the A-O-D-O method, tissues prefixed with aldehyde fixatives by perfusion are processed in the osmium maceration procedure, which is essential to avoid postmortem morphological alterations of tissues, especially of nerve tissues (Tanaka and Mitsushima, 1984). Using the maceration method, the 3D ultrastructure of cell organelles, such as the Golgi apparatus, mitochondria, ER, ribosomes on the rough ER, has been investigated using SEM in various tissues (Tanaka and Naguro, 1981; Tanaka and Mitsushima, 1984; Tanaka et al., 1986; Lea and Hollenberg, 1989; Lea et al., 1994; Ogata and Yamasaki, 1997). We have also applied the maceration method to elucidate the 3D ultrastructural characteristic of the Golgi apparatus and clarify the morphological diversity of this organelle in different types of cells (Koga and Ushiki, 2006; Koga et al., 2017a, see also Koga et al., 2017c for a review on this project). Thus, the osmium maceration method has broadened the possibilities for SE-mode SEM in the field of cell biology.

### Improvement and Further Perspectives of the Maceration Methods

We have established several useful techniques related to the osmium maceration method for expanding the potential of the maceration method for examining bio-specimens. To expose the subcellular structure on the freeze-cleavage surface of the cell, tissues should be immersed in diluted osmium solution for several days (usually 3–7 days) at 20°C in accordance with the original protocol of the osmium maceration method developed by Tanaka and colleagues (Tanaka and Naguro, 1981; Tanaka and Mitsushima, 1984). This conventional temperature condition is time-consuming, and the optimal period for adequate extraction of cytosol with diluted OsO_4_ is dependent on the cell type (Kendall et al., 1991; Weiser et al., 1991; Ogata and Yamasaki, 1997; Koga and Ushiki, 2006; Koga et al., 2020). To improve the efficiency of the osmium maceration procedure, we examined the relationship between the reaction temperature and time of the maceration procedure and verified that temperatures higher than the conventional 20°C accelerated this process (Koga et al., 2020). We concluded that a higher temperature condition, especially at 30°C and 40°C, significantly shortens the reaction time to 30 and 15 h, respectively, without the deterioration of the ultrastructure of the organelles. The modification of the osmium maceration method (i.e., the rapid osmium maceration procedure) also achieved successful observation of the 3D ultrastructure of cell organelles in various cell types, including nerve cells.

Although most studies using the osmium maceration method have been conducted in tissues *in vivo*, few studies have applied the method to free and cultured cells (Fukudome and Tanaka, 1986; Isola et al., 2009). For the freeze-cracking of cells before the osmium maceration procedure, cells should be embedded in materials that are resistant to the osmium maceration procedure with prolonged osmication using diluted OsO_4_ for several days. We used low-melting-point agarose as the embedding material and successfully observed the 3D ultrastructure of organelles in HeLa and human blood cells using SEM with the osmium maceration method (Koga et al., 2012). Agarose-embedded cells that are further embedded in epoxy resin permit comparisons between SEM findings and ultrathin images of resin-embedded sections. Thus, the combination of the osmium maceration method with agarose embedding can be applied widely to 3D morphological analyses of the subcellular structure in various types of cultured cells as well as leukocytes and microorganisms.

The osmium maceration method provides valuable information on the 3D ultrastructure of organelles. However, combining immunocytochemical techniques with the maceration method is difficult because the OsO_4_ used for fixation and the maceration procedure impair the antigenicity of the molecules in specimens, which has long been a critical unresolved issue. To solve this challenge, we newly developed an efficacious modification technique for the osmium maceration method that links the information on the relationship between the location of molecules with the 3D ultrastructure of organelles.

The cryo-sectioning technique introduced by Tokuyasu (1973, 1989) is a well-established technique that enables the cutting of ultrathin or semithin cryosections of tissues treated with a cryoprotectant, such as high-molar sucrose or a mixture of PVP with a high-molar sucrose solution, for preserving the ultrastructure and antigenicity of cells in a cryo-chamber attached to an ultramicrotome. The Tokuyasu method has been applied to immuno-EM using TEM of ultrathin cryosections labeled using immunogold techniques (Tokuyasu, 1980; Takizawa and Robinson, 1994) and to correlative light and electron microscopy (CLEM) using TEM (Takizawa et al., 1998; Takizawa and Robinson, 2003; see also Takizawa et al., 2015 for a review). CLEM is a key imaging technique that is based on the observation of target molecules tagged with fluorescent dyes or proteins, such as green fluorescent protein, at a high-resolution electron microscopic level; it is expected to bridge the resolution gap between LM and EM.

The combination of the osmium maceration method and the cryo-sectioning technique, a novel CLSEM established by our group, overcomes the drawback of the maceration method described above. The CLSEM is achieved by correlating the same area of immunofluorescence images of the cryosection with the SEM images of the adjacent face of the tissue block prepared using the osmium maceration procedure (Koga et al., 2015a). Using our CLSEM technique, we demonstrated the relationship between the localization of target molecules (e.g., the Golgi-associated protein, TGN38, and the representative ER chaperone, binding immunoglobulin protein, in anterior pituitary cells observed using immunofluorescence microscopy of the cryosection) and the 3D ultrastructure of organelles imaged using SEM of the tissue block face, adjacent to the section. Further applications of the combination of immunogold techniques with the maceration method will provide a more accurate and direct visualization of target molecules on osmium-macerated tissues, which will help to better understand neurological diseases and disorders that involve the morphological degeneration of organelles. Detailed information on the rapid osmium maceration method, the combination of the osmium maceration method and the agarose embedding technique, and CLSEM by combining the osmium maceration and Tokuyasu methods are available in our original article (Koga et al., 2012, 2015a, 2020).

### Semithin Section SEM: Section-Face Imaging of Semithin Sections Embedded in Resin

TEM of a single ultrathin section embedded in resin enables observations of the fine structure of tissues and cells. Although TEM provides invaluable information on the morphological feature of bio-specimens, ultramicrotomy is an essential technique for cutting resin-embedded tissues into ultrathin sections (80–100-nm thickness), which requires a high level of skill that is acquired with long-term training. To overcome these technical problems of TEM, we recently established a novel imaging technique termed semithin section SEM (i.e., section-face imaging of semithin sections) for acquiring ultrathin images that are comparable to those acquired using conventional TEM by observing resin-embedded semithin sections using BSE-mode SEM. It requires no expert techniques, such as ultramicrotomy (Koga et al., 2015b).

Recent instrumental improvements in the scanning electron microscope (e.g., field-emission guns, lenses, and signal detection systems) have allowed us to obtain information on resin-embedded ultrathin sections using BSE-mode SEM (Micheva and Smith, 2007; Micheva et al., 2010; Horstmann et al., 2012; Reichelt et al., 2012). Because the intensity of BSE signals is dependent on the average atomic number of the specimen composition, BSE-mode SEM of resin-embedded sections stained with heavy metals, such as osmium, uranium, and lead, can provide high-contrast profile images of organelles. Furthermore, the escape depth of BSE signals depends on the value of the accelerating voltage of the incident electrons; BSE signals escape from different penetration depths according to the value of the accelerating voltage. BSE-mode SEM under a low accelerating voltage allows the detection of BSE signals from near the surface area of the sections, whereas signals can be detected from the deeper regions of specimens by observing under a high accelerating voltage. To the best of our knowledge, BSE-mode SEM under a low accelerating voltage of 2–2.5 kV provides depth information at 80–100 nm from the surface of tissue sections embedded in resin, which corresponds to the thickness of ultrathin sections. This assumes that ultrathin images are obtained regardless of the thickness of resin-embedded sections using BSE-mode SEM under low accelerating voltage conditions. By exploiting the characteristics of BSE signals (i.e., the relationship between the escape depth of BSE signals and the accelerating voltage), we succeeded in obtaining ultrathin images with sufficiently high-resolution by observing semithin sections (500-nm to 1-μm thickness) on glass slides using BSE-mode SEM (Koga et al., 2015b). Thus, section-face imaging of semithin sections enables the acquisition of TEM-like images without ultramicrotomy. Conveniently, semithin section SEM can also be applied to specimens embedded in resin previously prepared for TEM and resin-embedded semithin sections that have been cut previously that have been stored in slide boxes.

London Resin (LR; London Resin Co. Ltd., Hampshire, UK) white resin is an alternative embedding medium to epoxy resin and is widely used for post-embedding immunohistochemistry and immuno-EM (Sakai et al., 2005; Watanabe et al., 2012). This resin with a stable electron beam and hydrophilic properties is preferred for CLSEM using BSE-mode SEM. The hydrophilic characteristic allows immunocytochemical reagents, such as antibodies, to penetrate resin-embedded tissue sections without requiring any etching treatment. We previously performed immunocytochemical correlation analysis between LM and BSE images of semithin sections embedded in LR white resin and localized the luteinizing hormone on secretory granules of gonadotropes in the rat anterior pituitary gland by superimposing the two images. Detailed information on the CLSEM using LR white resin can be found in our previous articles (Koga et al., 2015b).

### The Combination of the Cryo-Sectioning Technique With Section-Face Imaging

As described above, the Tokuyasu cryo-sectioning technique provides ultrathin and semithin cryosections of tissues that maintain their ultrastructure and antigenicity. By exploiting the merits of the Tokuyasu method, we recently established a novel CLSEM technique by combining the cryo-sectioning technique with section-face imaging (Kusumi et al., 2018; see also Koga et al., 2018 for a review on this technique). Both semithin cryosections were cut according to the Tokuyasu procedure and the thicker frozen tissue sections prepared using a conventional cryostat microtome had better antigen accessibility and were more suitable for immunostaining with a variety of commercially available primary antibodies than those of sections embedded in resins, such as LR white and epoxy resin. However, the ultrastructural preservation of intracellular structures in cryosections cut using the Tokuyasu method was considerably better than that using conventional cutting methods with a cryostat because a mixture of PVP and high molar sucrose in cryoprotectants in the Tokuyasu method contributes significantly to preserving the formation of ice crystals in tissues. As shown in Figures 7D,E, sufficient contrast of organelles was observed using BSE-mode SEM in the ultrathin sections of the resin-embedded semithin cryosection, even after the cryosection was immunocytochemically stained and viewed using a confocal laser scanning microscope (Figure 7A). Thus, our CLSEM technique can offer precise subcellular localization of target molecules labeled with fluorescence dyes and the corresponding ultrastructures of immunocytochemically labeled organelles.

Further applications of the CLSEM using FluoroNanogold (FNG), which combines two different probes, such as nanogold and fluorescence dyes (Nanoprobes, NY, USA), allow us to obtain more detailed information on the localization of target molecules imaged using LM at the EM level (Koga et al., 2015b; Kusumi et al., 2018). Molecules imaged as fluorescence signals using LM can be simultaneously observed as nanogold, which need to be previously treated with a gold or silver enhancement procedure to render them visible under SEM. Thus, the combination of Tokuyasu cryosectioning and section-face imaging will be widely applicable to biological and biomedical research using CLEM and immuno-EM.

### Serial Section SEM and the 3D Reconstruction Method

In contrast to section-face imaging, block-face imaging is a BSE-imaging technique that is essential for FIB-SEM and SBF-SEM and provides ultrathin information on resin-embedded tissue blocks using SEM. FIB-SEM and SBF-SEM provide serial tomographic images by repeating cycles of block-face elimination of resin-embedded specimens using a FIB instrument and microtome installed in scanning electron microscopes, respectively, to capture newly detected block-faces. Array tomography introduced by Micheva and Smith (2007) is a contrasting 3D imaging approach for obtaining sequential tomographic images of consecutive ultrathin sections on solid substrates, such as glass microscope slides or silicon wafers, using LM and/or SEM. Serial section SEM, a technique related to array tomography, is a specialized imaging technique that uses BSE-mode SEM of sequential sections. This novel 3D-SEM technique enables observation of the shape of entire organelles (e.g., the Golgi apparatus, mitochondria, and ER) that are spread widely throughout the cytoplasm by creating 3D reconstruction models from consecutive tomographic images of resin-embedded ultrathin sections obtained using BSE-mode SEM (Horstmann et al., 2012; Reichelt et al., 2012; Wacker et al., 2016). We examined the entire 3D shape of the Golgi apparatus in different cells, which included the secretory, epithelial, and nerve cells, and demonstrated the morphological diversity of this organelle using serial section SEM. The entire shape of the Golgi apparatus varies depending on the cell type; it is a large organelle that is located in the widest part of the cytoplasm and comprises a single uninterrupted network except for in Purkinje cells (Koga et al., 2016b, 2017b). Following these results, the next step is to investigate whether the discontinuity of the Golgi apparatus is specific to neurons. Further studies using serial section SEM will provide valuable information on the entire shape of the Golgi apparatus, which will accelerate accurate interpretations of the 3D structure of this organelle. We aim to elucidate the morphological diversity of the Golgi apparatus in various types of neurons, in addition to the Purkinje cell, using the novel 3D-SEM technique to solve the morphological mystery of the Golgi apparatus. Moreover, morphological studies based on direct 3D information obtained using high-resolution SE-mode SEM of osmium-macerated tissues combined with 3D reconstruction models generated from consecutive ultrathin sections captured using BSE-mode SEM will reveal neural tissues with complicated structures in three dimensions. Such investigations will provide valuable information on the entire shape of the Golgi apparatus and the functional significance of the peculiar 3D structure of this organelle in neurons.

### Advantages and Disadvantages of Serial Section SEM

Serial section SEM provides a wide observation field that is determined by the trimming area of the resin-embedded tissue blocks corresponding to the size of the serial sections embedded in resin, which can be adjusted liberally according to the purpose of the study. Serial section TEM, which is a classical and crucial 3D EM technique that is a similar 3D approach to the serial section SEM, has enabled the acquisition of 3D reconstruction images of organelles and neuronal structures (Dylewski et al., 1984; Tamaki and Yamashina, 1997; Ostroff et al., 2002; Knott et al., 2006; Kubota et al., 2009). In serial section TEM, consecutive serial ultrathin sections of tissues embedded in resin are mounted onto the limited space of single-slot grids, of which the area of each section is inevitably smaller than that of serial section SEM. Moreover, resin-embedded sections on single-slot grids should be supported by the membranes of conventional materials, such as collodion or formvar, which are fragile and unstable for electron beam irradiation. Thus, the distortion and/or disruption of sections disturb the generation of accurate 3D reconstruction models. In contrast, resin-embedded tissue sections on solid substrates, such as glass microscope slides that are coated with carbon or osmium, are stable for electron beam irradiation, although charging artifacts and thermal damage can occur during imaging using BSE-mode SEM of sections without conductive treatment.

Resin-embedded tissue sections on solid substrates can be stained with heavy metals, such as uranyl acetate and lead citrate, and is routinely performed in sections on grids for conventional TEM preparation, which, unlike SBF- and FIB-SEM, provides TEM-like images of ultrathin sections. Moreover, these sections allow immunocytochemical staining with various target molecules. Post-embedding immuno-labeling for serial section SEM has a considerable advantage over other 3D-SEM techniques that use block-face imaging. Hydrophilic resins, such as LR white resin, can be used instead of hydrophobic resins (e.g., epoxy resin) as the embedding material, which offers further opportunities for using immunocytochemical approaches with SEM (Micheva and Smith, 2007; Micheva et al., 2010; Oberti et al., 2011; Koga et al., 2017b).

Serial sections on solid substrates allow long-term storage in a desiccator, which enables repeated analyses of ROIs and other structures of interest previously observed using BSE-mode SEM. Thus, findings obtained *via* the generation of 3D models of structures of interest in consecutive tomographic images captured using serial section SEM can be revalidated, which is a significant process for morphological science research. In contrast, block-face resin-embedded tissues are destined to disappear due to milling and cutting in FIB-SEM and SBF-SEM, respectively. Furthermore, the most significant advantage of serial section SEM is that it allows the structures of interest for 3D reconstruction to be determined in advance before acquiring serial tomographic images.

Although serial section SEM has numerous advantages as described above, it also has several drawbacks. The z-resolution of the method corresponds to and is limited by the thickness of the serial sections, and it is considerably inferior to FIB-SEM, in which the milling pitch can be set to several nm per step. From our experience, we deem a section thickness of 80–100 nm is desirable for creating successful ribbons of serial ultrathin sections. The cutting and collection of ribbons demand a high level of skill and labor, and these processes are crucial for serial section SEM. The methods used are diverse and vary between researchers; moreover, a variety of techniques have been introduced by microscopists: a diamond knife with a jumbo boat (Micheva and Smith, 2007), diamond knives combined with custom-built devices (Horstmann et al., 2012; Wacker et al., 2016), and crane-like devices (Koike and Yamada, 2019). We established a new method for cutting and collecting ribbons of serial ultrathin sections by using a diamond knife and aluminum loops, respectively (Koga et al., 2016b), which allows stable cutting of over a thousand serial sections. The automated tape-collecting ultramicrotome is a device that is attached to an ultramicrotome that enables automatic collection of resin-embedded serial sections cut using a diamond knife on Kapton tape (Terasaki et al., 2013), which has been widely adapted to neuroscience research (Kasthuri et al., 2015; Morgan et al., 2016; see also review Kubota et al., 2018).

In serial section SEM, structures of interest are recorded by manual operation of BSE-mode SEM of consecutive serial sections on solid substrates, which, unlike FIB-SEM and SBF-SEM, is time-consuming and labor-intensive. However, automated capturing software is already in production, which will help solve this problem. We believe that it is important to highlight the advantages and disadvantages of each 3D-SEM method to ensure that the appropriate method is applied according to the morphological analysis target structure or study purpose.

## Conclusion

This article focused on our related SEM methods that use SE and BSE signals: the osmium maceration method, section-face imaging, and serial section SEM. We believe that these techniques provide useful information and allow comprehensive analyses of neural circuits. Recent instrumental advances in the scanning electron microscope have enabled imaging of specimens embedded in resin using BSE-mode SEM, which has broadened the possibilities of SEM in the biological and biomedical fields. Although the focus has largely been on novel SEM techniques using BSE signals (i.e., FIB-SEM, SBF-SEM, and serial section SEM), we cannot neglect other valuable techniques for SE-mode SEM (e.g., the conventional technique as well as methods for connective tissue removal, revealing the collagen fibrillar network, visualizing the microvascular circulation, and observing the subcellular structure) that provide useful information on the 3D structure of tissues and cells. Accurate comprehension of the ultrastructure of bio-specimens requires comparisons of tissue micrographs prepared using various morphological approaches. In the future, we aim to establish novel SEM methods by combining various morphology or biochemical techniques with our EM techniques, which will help clarify the 3D ultrastructure of neural structures as well as organelles.

## Author Contributions

DK and SK performed the experiments and wrote the original draft. TW reviewed and revised the manuscript. MS reviewed the manuscript. All authors contributed to the article and approved the submitted version.

## Conflict of Interest

The authors declare that the research was conducted in the absence of any commercial or financial relationships that could be construed as a potential conflict of interest.

## Publisher’s Note

All claims expressed in this article are solely those of the authors and do not necessarily represent those of their affiliated organizations, or those of the publisher, the editors and the reviewers. Any product that may be evaluated in this article, or claim that may be made by its manufacturer, is not guaranteed or endorsed by the publisher.
